# Interactive Effects of Dietary Starch Levels and Exogenous α-Amylase on Growth, Digestibility, and Metabolic Responses in *Channa striata* Juveniles

**DOI:** 10.3390/biology14091237

**Published:** 2025-09-10

**Authors:** Kaliyaperumal Sriranjani, Amit Ranjan, Albin Jemila Thangarani, Ambika Binesh, Mohamood Kavimugaraja, Subbiah Balasundari, Nathan Felix

**Affiliations:** 1 Institute of Fisheries Post Graduate Studies, Tamil Nadu Dr. J. Jayalalithaa Fisheries University, Vaniyanchavadi 603 103, Tamil Nadu, India; 2 Tamil Nadu Dr. J. Jayalalithaa Fisheries University, Nagapattinam 611 002, Tamil Nadu, India

**Keywords:** carnivorous fish, *Channa striata*, starch, amylase, growth performance, nutrient digestibility

## Abstract

A 70-day feeding trial was carried out to examine the effects of exogenous alpha amylase supplementation and different levels of starch on the growth performance, whole-body proximate composition, apparent nutrient digestibility, and digestive and metabolic enzyme activities of *Channa striata* juveniles. Nine semi-purified iso-nitrogenous (42%) and iso-lipidic (7%) diets containing three different levels of starch (*viz.* 10%, 20% and 30%) and amylase (0%, 0.05%, 0.1%) were formulated as C_10_A_0_, C_10_A_0.05_, C_10_A_0.1_, C_20_A_0_, C_20_A_0.05_, C_20_A_0.1_, C_30_A_0_, C_30_A_0.05_, and C_30_A_0.1_ (C-starch, A-amylase). A total of 405 *C. striata* juveniles of average weight (14.31 ± 0.1 g) were randomly assigned to 27 150 L capacity FRP tanks with 15 fish per tank following a 3 × 3 factorial design in triplicate with proper aeration. Final weight, weight gain (WG%), specific growth rate (SGR), feed conversion ratio (FCR), and protein efficiency ratio (PER) were significantly influenced (*p* < 0.05) by dietary starch and amylase supplementation as well as their interaction. The nutrient digestibility results revealed that the apparent digestibility coefficient of dry matter, crude protein, crude lipids, and carbohydrates improved significantly (*p* < 0.05) with higher amylase levels. There was no significant variation (*p* > 0.05) in the whole-body proximate composition of fish fed with different levels of starch and exogenous amylase supplementation. Amylase activity increased with higher dietary amylase levels; however, there were no significant differences in protease and lipase enzyme activity. Fish in the A_0.1_ treatment group had significantly higher (*p* < 0.05) hexokinase activity, which was significantly affected by exogenous amylase levels. AST and ALT activities in the serum were decreased (*p* < 0.05) at 0.1% amylase inclusion in the diet. From the present study, it is concluded that supplementation with exogenous alpha amylase has the potential to enhance starch utilization in *C. striata*. In particular, 0.1% amylase with 20% starch can significantly improve growth and nutrient utilization in *C. striata* juveniles without adverse effects.

## Simple Summary

*Channa striata* (striped murrel) is a highly valued freshwater fish for its taste, nutritional quality, and medicinal properties. However, as a carnivorous species, it has a limited capacity to utilize dietary carbohydrates efficiently, which restricts the use of carbohydrate sources in aquafeeds. To address these challenges, a 70-day feeding trial was conducted to evaluate the combined effects of different dietary starch levels (10%, 20%, and 30%) and exogenous α-amylase supplementation (0%, 0.05%, and 0.1%) on the growth, digestibility, and digestive and metabolic responses of *C. striata* juveniles. This study revealed that dietary α-amylase supplementation significantly improves the starch and dry matter digestibility, leading to improved growth performance and feed utilization efficiency of *C. striata*.

## 1. Introduction

In recent years, global aquaculture has witnessed substantial growth, reaching 130.9 million tons in 2022 [1] and offering crucial income and employment in many developing nations [2]. Similarly, the Indian aquaculture industry has emerged as one of the fastest-growing sectors worldwide owing to the production of aquatic species. Among murrel species, *Channa striata* (Bloch, 1793) is a highly valued freshwater fish [3,4]. This high economic value stems from desirable flesh quality and strong market demand [5]. *Channa striata* is a carnivorous, obligate air-breathing fish commonly known as the murrel or striped snakehead, and it possesses several traits that make it an ideal candidate for aquaculture. These include fast growth rate, good flesh quality, ease of handling, and remarkable tolerance to suboptimal water conditions [6]. In addition to its value as a food fish, recent studies have highlighted its medicinal potential, particularly in relation to the therapeutic properties of its flesh and body extracts [7].

The rapid growth and high metabolic activity of *C. striata* demand a substantial energy supply. In many fish species, proteins are often utilized as a key energy source, which may compromise their role in tissue development and growth. However, research has demonstrated that incorporating digestible carbohydrates into the diet can effectively spare proteins for anabolic processes [8]. Traditionally, fishmeal (FM) has served as the principal protein source in aquafeeds, playing a vital role in supporting rapid growth and overall physiological health [9]. To reduce feed costs, it is essential to enhance the utilization of non-protein energy sources, particularly carbohydrates, thereby preserving dietary protein for growth-related functions [10].

The inclusion of carbohydrates in aquafeeds supports sustainable aquaculture by reducing nitrogen load and providing metabolic intermediates. Balanced carbohydrate levels lower feed costs, improve pellet quality, and enhance fecal consistency for waste removal [11]. Carbohydrates serve as non-protein energy sources and are valued for their economic benefits in aquafeed formulations [12]. In fish, carbohydrates are mainly stored as glycogen in the liver and muscle tissues and act as a readily accessible energy reserve. These stored carbohydrates participate in several metabolic processes to fulfil an organism’s energy needs, such as gluconeogenesis, glycolysis, the pentose phosphate route, the tricarboxylic acid (TCA) cycle, and glycogen production [13].

Carnivorous fish species exhibit a limited capacity to efficiently metabolize dietary carbohydrates, a limitation largely attributed to digestive constraints and suboptimal regulation of glucose homeostasis at both hormonal and metabolic levels [14]. At moderate inclusion levels, carbohydrates can play a beneficial role by sparing protein and improving growth performance [15]. When supplied in excess, these carbohydrates exceed the limited metabolic handling capacity of carnivorous fishes, leading to impaired growth, reduced feed conversion efficiency, decreased protein utilization, and hepatic lipid accumulation, which can ultimately cause fatty liver syndrome and compromise liver function [16]. The extent to which carbohydrates are utilized by fish depends on several factors, such as species-specific metabolic capacity, the type and source of carbohydrates, processing methods, and dietary inclusion levels [17,18,19]. Subsequent research has indicated that exceeding certain carbohydrate threshold levels can be detrimental to fish. For instance, Marandel et al. [20] observed that dietary carbohydrate levels above 20% in rainbow trout (*Oncorhynchus mykiss*) resulted in sustained hyperglycemia, immune suppression, and tissue damage. Feeding fish with high carbohydrate levels has been associated with several negative physiological outcomes, including elevated blood glucose levels, excessive hepatic glycogen accumulation [21,22], impaired growth [19], reduced flesh quality [23], and weakened immune responses [24,25,26]. Previous studies have also reported that excessive carbohydrate intake leads to growth retardation and metabolic imbalances.

As a result, the incorporation of exogenous digestive enzymes into aquafeeds has received growing attention for its potential to enhance carbohydrate digestion and utilization. This strategy, which has long been established for monogastric livestock nutrition, particularly in poultry and swine diets [27,28,29], is now increasingly being explored in aquaculture. The incorporation of exogenous enzymes has featured as a valuable strategy for promoting sustainable aquaculture practices [30]. Numerous studies have shown that enzymatic pretreatment of plant-based feed ingredients in fish can significantly improve nutrient digestibility and growth by reducing anti-nutritional factors and non-starch polysaccharides [31,32,33]. Exogenous enzymes improve the digestion capacity of fish by increasing the activity of endogenous enzymes. This leads to better utilization of feed, resulting in improved growth performance [34,35]. Supplementation of alpha-amylase in fish diets has led to significantly improved starch utilization and glucose metabolism in *Labeo rohita* [36], regulation of postprandial blood glucose levels in fish, starch digestibility in silver perch [37], increased apparent nutrient digestibility in rainbow trout [38], improved fish immunity in striped catfish [39], and enhanced feed utilization rate in Atlantic salmon [40].

Despite these promising findings, most studies have focused on multi-enzyme complexes, and there remains a lack of information on the specific role of α-amylase as a single supplement, particularly in carnivorous fishes that have inherently low carbohydrate utilization capacity. Addressing this gap, the present study was designed to evaluate the combined effects of varying dietary starch levels (10%, 20%, and 30%) and different levels of α-amylase supplementation (0%, 0.05%, and 0.1%) on growth performance and nutrient utilization of *C. striata* juveniles.

## 2. Materials and Methods

### 2.1. Experimental Setup

The experiment was conducted in the wet laboratory of the Institute of Fisheries Post Graduate Studies, Chennai, India, for 10 weeks in a 150 L capacity FRP tank. A total of one thousand fish were procured from CR Aqua Tec Pvt Ltd., Hatchery, Udipalya, Bangalore, India, and were acclimatized for 2 weeks with a commercial diet (Nutrila™ Growel Feeds Pvt, Ltd., Singarayapalem, Andhra Pradesh, India CP-42%, CL-7%). After 2 weeks of acclimation, 405 fish (average wt. 14.31 ± 0.1 g) were randomly assigned to 27 150 L capacity FRP tanks, with 15 fish per tank. The experimental tanks were arranged sequentially from T1 to T9 and provided with a uniform water supply and aeration. The treatments were executed in triplicate (n = 3) using a 3 × 3 factorial design. At the start and end period of the experimental trail, the individual fish were weighed. The fish were fed thrice daily at 9:30 AM, 1:30 PM, and 5:30 PM until apparent satiation. Uneaten feed and fecal matter were removed daily by siphoning during a 50% water exchange, carried out prior to the first feeding at 9:30 AM. Fish were kept under a constant photoperiod (12 h light/12 h dark). Regarding water quality parameters, the pH ranged from 7.5 to 8.0 while the temperature remained between 27 and 29 °C. The DO level fluctuated between 6 and 8 mg/L. Total ammonia nitrogen levels were maintained below 0.9 mg/L. A commercial water quality test kit (Bionix freshwater master kit, Kolkata, India) manufactured by R.S. Fish Farm, Kolkata, India, was used to analyze all water quality parameters.

### 2.2. Experimental Diet Preparation

Nine iso-nitrogenous (42% crude protein) and iso-lipidic (7% crude fat) experimental diets were prepared, incorporating three levels of dietary starch (10%, 20%, and 30%) combined with three levels of alpha-amylase supplementation (0%, 0.05%, and 0.1%). The nine treatment groups were labelled as C_10_A_0_, C_10_A_0.05_, C_10_A_0.1_, C_20_A_0_, C_20_A_0.05_, C_20_A_0.1_, C_30_A_0_, C_30_A_0.05_, and C_30_A_0.1_, representing combinations of dietary starch (C) and amylase (A) levels.

Experimental diets were formulated using different ingredients, as shown in Table 1. Fish meal, acetes meal, and fish protein hydrolysate were used as protein sources, whereas fish oil and soybean oil were used as lipid sources and corn starch was used as a carbohydrate source. Cellulose was used as a filler and carboxymethyl cellulose (CMC) was used as a binder. All ingredients were ground well and weighed according to the requirements as outlined in Table 1. A small amount of water was added to obtain a consistent dough, which was then steam-cooked in a pressure cooker for 10 min. After cooling to room temperature, additional components such as fish oil, soybean oil, vitamin–mineral premix, vitamin C, tryptophan, and butylated hydroxytoluene (BHT) were incorporated. The required amount of thermostable alpha-amylase (sources: salmonella *E. coli*; 520,000 U/g) dissolved in 25 mL of water was added at this stage. Chromic oxide was added to the mixture as an inert marker to analyze the apparent digestibility coefficient. The final dough was fully mixed and run through a pelletizer fitted with a 2 mm die to produce wet pellets. These were then dried in a hot air oven at 40 °C to lower the moisture content and prevent fungal growth. The formulated feeds were stored at −20 °C until further use.

### 2.3. Proximate Composition

At the end of the feeding trial, six fish were randomly selected from each tank. These fish were then pooled based on the treatment groups to evaluate whole-body proximate composition. The standard techniques described by [41] were used to determine the proximate composition of the fish samples and experimental diet. A hot air oven (Equitron, India) was used to dry the samples at 100 ± 2 °C until a consistent weight was recorded in order to assess the moisture content. The crude protein was analyzed (N × 6.25), following acid digestion with a Foss™ Digestor™ 2508 (Foss Tecator line, Foss India Pvt. Ltd., Mumbai, India) and distillation with a Foss™ Kjeltec™ 8100 system, and titration was used to quantify the nitrogen content. Crude lipids were analyzed following petroleum ether extraction using the Soxhlet technique and SOCS Plus TM equipment (Pelican, Chennai, India). Total ash content was determined by burning the samples in porcelain crucibles at 550 °C for 6 h in a muffle furnace (Hasthas, Chennai, India). Crude fiber in the diet was analyzed using the Fiber Cap method (FIBRAPLUS, Pelican, India). A bomb calorimeter (IKAC 3000) (IKA-Werke GmbH & Co. KG, Breisgau, Germany) was used to analyze the gross energy content of the experimental diet.

### 2.4. Growth Parameters

After completion of the feeding trial, bio-growth parameters such as growth performance, weight gain (WG), specific growth rate (SGR), feed conversion ratio (FCR), and protein efficiency ratio (PER) were evaluated using the formulas described below [42,43].

Weight gain %

WG (%) = [final weight (g) − initial weight(g)]/initial weight (g) × 100

Specific growth rate (SGR)

SGR = Ln (Final weight) − Ln (initial weight)/Number of days × 100

Feed conversion ratio (FCR)

FCR = Feed given (g dry weight)/body weight gain (g wet weight)

Protein efficiency ratio (PER)

PER = weight gain (g)/protein fed (g).

### 2.5. Sample Collection

Prior to sampling, the fish were anesthetized with 100 mg/L tricaine methane sulfonate (MS-222, Sigma-Aldrich, St. Louis, MO, USA). Then, blood samples were collected from three fish per treatment by puncturing the caudal vein using a No. 23 gauge syringe that had been pre-rinsed with 2.7% EDTA to prevent clotting. The drawn blood was transferred into Eppendorf tubes and kept in a slanting position; after the coagulation of blood, it was centrifuged for ten minutes at 3000 rpm to extract the clear serum, which was then stored at −20 °C for further biochemical analysis. Tissue samples from the intestine and liver were obtained from the same fish used for the blood collection. These tissues were immediately homogenized in ice-cold 0.25 M sucrose solution using a mechanical homogenizer to preserve enzyme integrity. Following centrifugation of the homogenates at 5000 rpm for 10 min at 4 °C, the supernatants were collected and kept at −80 °C until further enzymatic analysis. The soluble protein content in the enzyme extract was quantified using the Bradford method (1976) with bovine serum albumin as the standard, and readings were taken at 595 nm using an ELISA Microplate Reader (BioTek Epoch 2, Agilent Technologies, Chennai, India). Absorbance was measured using a Cary 60 UV-Vis spectrophotometer (Agilent Technologies, Santa Clara, CA, USA), and the results were expressed as specific enzyme activity (U/mg protein and U/L).

### 2.6. Digestive Enzyme Assay

#### 2.6.1. Protease

The casein hydrolysis technique outlined by [44] was used to measure total protease activity. Tissue homogenate and a 1% casein solution made in 0.05 M Tris-phosphate buffer (pH 7.8) were combined for the experiment, which was then incubated for ten minutes at 37 °C. Then, 10% trichloroacetic acid (TCA) was added to stop the enzymatic process. Whatman No. 1 filter paper was used to filter the mixture, and the absorbance of the filtrate was measured at 280 nm. Under the specified assay conditions, one unit of protease activity was defined as the quantity of enzyme that produced acid-soluble fragments equal to one micromole of tyrosine per minute.

#### 2.6.2. Lipase

Lipase activity was measured using an olive oil emulsion as the substrate, in accordance with the procedure outlined in [45]. Measures of 1 mL of the sample homogenate, 2 mL of olive oil emulsion, 0.5 mL of phosphate buffer (pH 7.0), and distilled water were combined to start the reaction. This combination was then incubated for 24 h at 37 °C. Following incubation, the reaction mixture was supplemented with 3 milliliters of 95% alcohol and a few drops of phenolphthalein. The solution was titrated with 0.05 N NaOH until the endpoint was indicated by a constant pink shade.

#### 2.6.3. Amylase

A starch solution was used from a prepared phosphate buffer (pH 6.9) as the substrate, and amylase activity was determined using the technique outlined in [46]. The substrate and 0.1 mL of enzyme extract were mixed together for the experiment, which was then incubated for four minutes at 95 °C. The mixture was placed in a boiling water bath for five minutes after two milliliters of dinitro salicylic acid (DNS) to halt the process. Five milliliters of distilled water was added to the solution after it had cooled down. The absorbance was measured at 540 nm. Using a maltose standard as a reference, amylase activity was calculated as the quantity of maltose emitted per min at 37 °C.

### 2.7. Metabolic Enzyme

The activities of key protein metabolism enzymes, AST (aspartate aminotransferase) and ALT (alanine aminotransferase), were analyzed in fish serum samples using SGOT and SGPT diagnostic kits from Erba Diagnostics (Mannheim, Germany), following the instruction by manufacturers. Absorbance was measured at 340 nm, and the activity was expressed as units per liter (U/L).

Hexokinase (HK; E.C. 2.7.1.1) activity was measured using a technique developed by [47]. The reaction cocktail consists of 50 mM of glucose, 200 mM of MgCl_2_, Tris–HCl buffer, and 30 mM of ATP. The pH was adjusted to 7.6 at 30 °C. The final reaction mixture contained 500 U/mL of glucose-6-phosphate dehydrogenase (G6PDH) and 1 mM of β-NADP. The quantity of enzyme required to phosphorylate 1.0 micromole of D-glucose per minute at 30 °C was considered to be one unit of HK activity.

### 2.8. Collection of Fecal Matter

The fish were fed with the respective experimental diet three times daily at 9:30 AM, 1:30 PM, and 5:30 PM until apparent satiation. Following the method as described by [48], fecal matter collection began after a week of acclimatization to the experimental diet. To maintain clean tank conditions and remove any uneaten feed and waste, the tanks were siphoned for one hour after each feeding session. Fecal samples were collected one hour after feeding, twice a day at 10:30 AM and 2:30 PM, for a period of last 15 days. These samples were then pooled based on the treatment groups, dried in a hot air oven, and stored properly until further analysis.

#### Estimation of Digestibility

Chromic oxide (Cr_2_O_3_), an inert and indigestible marker, was uniformly dry-mixed with all other ingredients at a concentration of 5 g per kilogram of feed to calculate the apparent digestibility coefficient (ADC). The chromium content in both the diets and the collected fecal samples was analyzed using an inhouse SOP with an ICP-OES/MS instrument.

The ADC was determined using the following formula:

(%) ADC_diet_ = 100 − 100 [(C_td_∕N_td_) × (N_fe_∕C_fe_)]

where N_td_ and N_fe_ are the concentrations of nutrients in the treatment diet and feces, respectively; and C_td_ and C_fe_ stand for chromic oxide concentrations in the treatment diet and feces, respectively.

### 2.9. Statistical Analysis

All statistical data were analyzed using IBM SPSS version 27 (SPSS, Chicago, IL, USA). The effects of dietary starch, amylase level, and their interaction were determined via two-way analysis of variance (ANOVA). To analyze the significant difference among the treatment groups, one-way ANOVA was also performed. When significant effects were observed, post hoc comparisons were conducted using Duncan’s Multiple Range Test (DMRT), and the results were expressed as mean ± standard error (SE). Differences between means were considered statistically significant at *p* < 0.05.

## 3. Results

### 3.1. Growth Performance and Nutrient Utilization

The growth performance and nutrient utilization of *C. striata* fed diets formulated with different levels of carbohydrates and exogenous alpha-amylase are shown in Table 2. Dietary starch and amylase level and their interaction significantly (*p* < 0.01) affected the final weight, WG, SGR, FCR, and PER of *C. striata*. Significantly higher final weight, weight gain, SGR, and PER values were observed at the 20% starch inclusion level and 0.1% amylase level. A significantly lower (*p* < 0.05) feed conversion ratio (FCR) was observed at the 20% starch level and 0.1% amylase supplementation level.

### 3.2. Whole-Body Proximate Composition

The whole-body proximate composition of *C. striata* fed diets formulated with different experimental diets is shown in Table 3. The dietary starch and amylase level and their interaction had no significant (*p* > 0.05) effect on the crude protein, moisture, and total ash content of *C. striata* fed with different experimental diets. Significantly higher crude protein and crude lipid were observed at a 20% starch inclusion level and 0.1% amylase level. Significantly (*p* > 0.05) lower crude protein and crude lipid observed at 10% starch and 0% amylase levels. No significant variation was observed in dietary starch and amylase and their interaction with the total ash and moisture content of *C. striata*.

### 3.3. Apparent Digestibility Coefficient

The dry matter, crude protein, and crude lipid apparent digestibility coefficients (ADC) of *C. striata* fed diets formulated with different levels of starch and amylase are shown in Table 4. The dry matter and carbohydrate digestibility were significantly (*p* < 0.05) affected by different levels of starch, amylase, and their interaction. Dry matter and carbohydrate digestibility were significantly (*p* < 0.05) lower at 30% starch inclusion level whereas no significant differences were observed at 10% and 20% starch levels. Amylase supplementation improved digestibility, which was significantly higher at a 0.1% inclusion level. Crude protein digestibility was not affected by starch levels, amylase levels, or with their combination (*p* > 0.05). Crude lipids were affected by starch levels and the interaction of starch and amylase; however, amylase alone had no effect on crude lipid digestibility in diets with different starch and amylase levels.

### 3.4. Digestive Enzyme Activity

Digestive enzyme activities, such as amylase, protease, and lipase, in the intestine of *C. striata* fed diets formulated with varying levels of starch and amylase are displayed in Table 5. Dietary starch and amylase levels did not significantly affect protease and lipase activity (*p* > 0.05). However, amylase activity was increased (*p* < 0.05) with higher dietary amylase level and peaked at 20% starch with 0.1% amylase level. Protease and lipase activity showed a decreasing trend with increasing levels of amylase in the diet. Different levels of starch and amylase and their interactions significantly (*p* < 0.05) affected the amylase activity in *C. striata* fed with different levels of starch and amylase in their diets. However, different levels of starch and amylase did not significantly affect the activity of protease enzyme; however, no significant effects were found for the combination of starch and amylase. Lipase activity was not significantly different (*p* > 0.05) at different starch levels, amylase levels, or their combinations.

### 3.5. Metabolic Enzyme Activities

The activities of metabolic enzymes, including Hexokinase, AST, and ALT, in the liver of *C. striata* fed diets formulated with different levels of carbohydrates and amylase are displayed in Table 6. Hexokinase activity was significantly affected (*p* < 0.05) by 0.1% amylase supplementation compared to both 0% and 0.05% amylase supplementation. However, the main effect of the dietary starch level alone and the interaction between starch and amylase were not found to be statistically significant (*p* > 0.05). AST activity was significantly influenced (*p* < 0.05) by both dietary starch level and amylase level; however, their interaction had no effect on AST activity (*p* > 0.05). A higher level of AST was observed in the diet including 30% starch without amylase supplementation. ALT levels also showed a similar trend with starch, amylase, and their interaction effects. A higher level of ALT was observed in the diet including 30% starch without amylase supplementation.

## 4. Discussion

The present research was focused on assessing the effects of different levels of dietary starch (10%, 20%, and 30%) in combination with different concentrations of amylase (0%, 0.05%, and 0.1%) on the growth performance, nutrient utilization, and enzyme activity of *C. striata*. Findings from this experiment revealed that the inclusion of amylase alongside an optimal level of dietary starch significantly enhanced fish growth. Remarkably, fish that were given a diet with 20% starch and 0.1% amylase showed the most significant improvements in weight gain (WG), final body weight, and specific growth rate (SGR), while also achieving the lowest feed conversion ratio (FCR). This indicates that dietary starch up to 20% combined with 0.1% amylase can substantially improve growth performance of *C. striata*. These findings align with those of [49], who reported increased WG and SGR in corn-based diets enriched with exogenous α-amylase. Similarly, [33] demonstrated that the inclusion of a mixture of multi-enzyme (xylanase, cellulase, glucanase, phytase and pentosanase) complexes improved both development and FER in *Lateolabrax japonicus*. Other research by [50] showed that supplementation of alpha amylase improves growth and feed conversion ratio in *Oreochromis niloticus*, as well as feed efficiency and flesh quality in Atlantic salmon (*Salmo salar*) [40]. A multi-enzyme complex including xylanase, neutral protease, and β-glucanase was also found to considerably increase the growth and feed conversion of hybrid tilapia (*O. niloticus* × *O. aureus*) by [35]. Additionally, it has been demonstrated that adding (NSP) non-starch-polysaccharide-degrading enzymes to plant-based feed ingredients may mitigate their anti-nutritional effects, improving growth in species such as tilapia [51], large yellow croaker [52], and Japanese seabass [33]. The observed improvements in growth performance likely result from enhanced nutrient breakdown and absorption due to increased digestive enzyme activity. Exogenous enzyme supplementation not only avoids suppressing endogenous enzyme activity but may actually stimulate it, leading to better nutrient utilization [35,53,54,55]. The superior performance observed in the 20% starch and 0.1% amylase group can be attributed to multiple interrelated mechanisms. Exogenous α-amylase supplementation likely enhances starch hydrolysis, thereby accelerating glucose release in the intestinal lumen. The increased glucose availability provided a readily utilizable energy source, which, in turn, may have supported greater hepatic glycogen deposition and improved the energy supply for somatic growth. This response could be linked to intestinal enzymatic adaptation, as evidenced by elevated amylase and hexokinase activities, which collectively facilitate more efficient carbohydrate digestion and metabolic utilization. These findings are consistent with earlier reports on rainbow trout (*Oncorhynchus mykiss*) [56] and Atlantic salmon [40], reinforcing the idea that exogenous enzymes can effectively counterbalance the limitations of plant-based diets in aquafeeds and enhance the overall growth response and feed efficiency in carnivorous fish.

In the present study, we observed a clear positive influence of exogenous alpha amylase supplementation on nutrient digestibility in *Channa striata*. Notably, intestinal amylase activity was significantly elevated in the group supplemented with 0.1% α-amylase compared to all other treatment groups. This enhanced enzymatic activity corresponds to improved digestibility of both carbohydrates and dry matter, indicating that amylase-mediated starch breakdown was the primary cause of the increase in nutritional absorption. This improvement in dry matter and carbohydrate digestibility might be driven by enhanced amylase mediated starch breakdown, which, in turn, allows nutrients to be utilized more efficiently. This was supported by the significantly higher intestinal amylase activity observed in the amylase supplemented groups, suggesting that exogenous α-amylase not only directly contributed to starch hydrolysis but also stimulated endogenous enzyme activity. As a result, glucose that would otherwise remain inaccessible due to the complex structure of starch became available, further enhancing nutrient absorption and overall digestibility. Our findings are supported by [57], who reported that dietary supplementation with exogenous α-amylase could enhance endogenous enzyme activity in fish, thereby improving the overall nutrient utilization. Specifically, in the current study, 0.1% α-amylase supplementation significantly increased the apparent digestibility coefficient (ADC) of carbohydrates. Given that starch molecules bind to the enzyme’s active site and are hydrophilic in nature [58], it is likely that the presence of α-amylase facilitated more efficient hydrolysis of dietary carbohydrates, as also suggested by in vitro digestion trials. Previous research has similarly highlighted improvements in nutrient digestibility with enzyme supplementation. For example, ref. [59] reported enhanced carbohydrate digestibility in Indian major carp, such as Catla and rohu, when fed gelatinized corn diets supplemented with amylase. Comparable results were found in rainbow trout and silver perch fed diets based on dehulled lupins, where the addition of α-galacosidase and natustarch (α-amylase) significantly improved the ADC [17,34]. Further evidence by [60] showed that the addition of β-glucanase, xylanase, and protease to diets rich in plant proteins, such as soybean, sunflower, and rapeseed, moderately enhanced the digestibility of sunflower and rapeseed meals, whereas β-glucanase particularly improved the digestibility of soybean-based diets. Similarly, ref. [61] found that xylanase supplementation at 3750 U/kg elevated intestinal enzyme activities such as lipase and chymotrypsin in fish. Interestingly, the positive impact of α-amylase extends beyond aquaculture. In poultry, the use of α-amylase-producing bacterial cultures has been shown to enhance serum and intestinal amylase activity in broiler chickens [62], reinforcing our observations in *C. striata*. These findings underscore the role of α-amylase in improving carbohydrate digestibility and energy availability, which, in turn, supports improved nutrient utilization and growth performance in *Channa striata*.

Several physiological outcomes, such as hunger control, development, survival, and reproductive success, are significantly influenced by the whole-body proximate composition of fish [63]. It also serves as an indicator of the energy density of fish, which is typically estimated based on the standard caloric values of proteins and lipids [64]. In the current research, variations in dietary starch levels and amylase supplementation had no significant effects on the whole-body composition of *C. striata*, including moisture, total ash, crude lipid, and crude protein content. This outcome suggests that nutrient retention in *C. striata* was relatively stable and was primarily influenced by the overall dietary nutrient profile rather than by the inclusion of enzymes or starch variation. Similar findings have been reported previously. For instance, ref. [65] found that supplementation with phytase and a commercial enzyme blend (including β-glucanase, xylanase, and protease) had insignificant effects on the proximate body composition of *Salmo salar*. Likewise, ref. [35] observed no substantial changes in moisture, protein, lipid, or ash content in *Oreochromis niloticus* × *O. aureus* fed diets enriched with enzyme complexes. Further supporting these results, ref. [66] reported that exogenous enzyme inclusion had negligible effects on the whole-body composition of fish. More recently, ref. [67] reported that hybrid grouper (*E. fusiguttatus* × *E. lanceolatus*) fed diets with different carbohydrate-to-lipid ratios showed no significant differences in crude protein, lipid, moisture, or ash content. These findings align with the present study, indicating that while enzymes may enhance nutrient digestibility and growth, they do not markedly alter the proximate composition of the fish body under standard feeding conditions, and fish can channel excess nutrients into energy metabolism rather than altering body composition.

It is well established that the digestive tracts of most fish species contain enzymes responsible for the metabolism of macronutrients, including proteins, lipids, and carbohydrates such as glycogen. The activities of these intestinal enzymes are positively correlated with the efficiency of nutrient digestion and absorption [68]. In this research analysis, the intestinal amylase activity of *Channa striata* significantly elevated with the different inclusion of dietary α-amylase, particularly when fish were fed a diet containing 20% dietary starch supplemented with 0.1% amylase. These results suggested that endogenous enzyme secretion or activity may be stimulated by exogenous enzyme supplementation. Generally, digestive enzyme activity is closely linked to fish growth [69]. Similar outcomes have been reported in other species; for instance, ref. [35] observed significantly improved amylase and protease activities in the intestine and hepatopancreas of tilapia with increased dietary enzyme supplementation. Likewise, increased dietary starch improved intestinal amylase activity and growth in gibel carp and grass carp [70], rohu [71], and African catfish [72] up to optimal inclusion levels. Ref. [73] discovered that adding xylanase to plant-protein-based diets enhanced trypsin, chymotrypsin, lipase, and amylase activity in carp. A similar trend was observed in white seabream [74], where supplementation of low fishmeal diets with NSP-degrading enzymes increased intestinal lipase and amylase activities. In tilapia (*Oreochromis niloticus*), the addition of an exogenous enzyme complex comprising xylanase, β-glucanase, and neutral protease significantly elevated the amylase and protease levels [35], while another study by [75] found that cellulase supplementation improved amylase and protease activities, though not lipase in grass carp. Additionally, ref. [51] also confirmed that NSP enzyme supplementation increased amylase activity without affecting protease activity. Excessively high dietary carbohydrate levels can suppress intestinal amylase activity in cultured fish species [76], even though the effectiveness of carbohydrate hydrolysis by enzymes relies on substrate concentration [77]. This was also reflected in the present study, where fish fed a high-starch diet (30%) without alpha amylase supplementation showed negative effects on intestinal amylase activity and also decreased the protease and lipase activity, which might be attributed to enhanced carbohydrate utilization in the presence of exogenous α-amylase, which supplied sufficient energy, thereby reducing the need for protein and lipid to be catabolized for energy.

Hexokinase (HK) is a pivotal enzyme in the glycolytic pathway that catalyzes the conversion of glucose to glucose-6-phosphate. This reaction marks the first committed forward move in glycolysis and is essential for controlling cellular glucose metabolism [78,79]. Fish species, particularly carnivorous species, have often been described as glucose-intolerant or exhibiting “diabetic-like” traits because of their limited ability to maintain stable blood glucose levels. This metabolic constraint is frequently attributed to suboptimal insulin secretion and reduced activity of hepatic glycolytic enzymes [80,81,82]. Dietary starch enhances glucose availability, which stimulates hexokinase activity and promotes glycolysis; however, when intake exceeds the tolerance of the species, metabolic overload can impair hepatic health and lead to insulin resistance, hyperglycemia, and non-alcoholic fatty liver disease, reflecting the inherent glucose intolerance of carnivorous fish [83]. These metabolic limitations have been supported by earlier studies on salmonids and cyprinids, where hexokinase activity showed minimal responsiveness to elevated dietary starch levels [78,84]. Similarly, ref. [85], examining HK activity in Atlantic salmon, reported no significant changes with increasing dietary starch level. However, contrasting evidence from [19] demonstrated that when dietary starch levels increased from 0 to 16.8%, the hepatic HK activity of juvenile golden pompano (*Trachinotus ovatus*) increased significantly, after which enzyme activity plateaued. In present research, varying dietary starch levels (10%, 20%, and 30%) did not significantly influence hepatic HK activity in *Channa striata*. However, there was a clear upward trend in HK activity with incremental supplementation of exogenous α-amylase (0%, 0.05%, and 0.1%). This observation suggests that although dietary starch alone did not stimulate hexokinase, the enhanced breakdown of starch by α-amylase may have increased glucose availability in the intestine, indirectly promoting HK activity. The differing outcomes between this and previous studies could be attributed to species-specific variations in glucose metabolism and the regulatory mechanisms governing enzyme expression. These results highlight the potential role of exogenous enzymes in improving nutrient digestion and modulating key metabolic pathways in carnivorous fish like *C. striata*.

The physiological and biochemical parameters of fish blood are strongly influenced by the dietary nutrient composition, making them valuable indicators of metabolic status, nutritional balance, and overall physiological health [86]. Among these, plasma alanine aminotransferase (ALT) and aspartate aminotransferase (AST) are commonly used biomarkers of hepatic function, with higher levels indicating hepatic stress or damage [87]. The liver, a central organ in immune function and metabolism, is particularly susceptible to dietary imbalances. Excessive carbohydrate intake has been reported to induce metabolic disturbances and structural liver damage in various fish species [49,88]. An increase in serum ALT and AST levels typically occurs when the liver cell membranes are compromised, allowing these intracellular enzymes to leak into the bloodstream. This leakage may also reflect increased enzyme synthesis in response to liver tissue injury [89]. Hence, serum ALT and AST levels serve as sensitive indicators for evaluating liver health in fish [90]. Oxidative damage and antioxidant imbalances are largely caused by free radicals, which are created during lipid metabolism by organisms that require oxygen [91]. Under normal physiological conditions, ALT and AST levels in the blood remain at a reduced level. However, structural damage to the liver cells leads to a notable rise in transaminase activity. In the present study, juvenile *Channa striata* fed diets with increased starch levels showed elevated serum AST and ALT activity. Interestingly, a progressive decrease in these metabolic enzyme levels was also observed with increasing supplementation of exogenous α-amylase (0%, 0.05%, and 0.1%). Despite this trend, the combined inclusion of starch and α-amylase did not appear to negatively affect liver health. Rather, this suggests that *C. striata* exhibits good physiological adaptability to dietary starch when supplemented with α-amylase, potentially reflecting improved nutrient metabolism without signs of hepatic stress.

## 5. Conclusions

In this study, it was evident that dietary starch at 20% and alpha amylase supplementation at 0.1% significantly improved the growth performance, apparent carbohydrate digestibility, amylase activity, and hexokinase level and reduced the activity of stress-regulated metabolic enzymes (AST and ALT). Different levels of starch and alpha amylase did not have any significant effects on the body composition or protease and lipase activity in *C. striata* juveniles. From the present research, it is concluded that the supplementation of 0.1% amylase to a 20% starch-based diet significantly improves the growth performance and nutrient utilization of *C. striata* juveniles.

## Figures and Tables

**Table 1:** Feed composition and proximate composition of experimental diets.

Feed Composition	Diets 1	Diets 2	Diets 3	Diets 4	Diets 5	Diets 6	Diets 7	Diets 8	Diets 9
	C_10_A_0_	C_10_A_0.05_	C_10_A_0.1_	C_20_A_0_	C_20_A_0.05_	C_20_A_0.1_	C_30_A_0_	C_30_A_0.05_	C_30_A_0.1_
Fish meal	50	50	50	50	50	50	50	50	50
Acetes meal	6.78	6.78	6.78	6.78	6.78	6.78	6.78	6.78	6.78
Fish protein hydrolysate	5	5	5	5	5	5	5	5	5
Starch	10	10	10	20	20	20	30	30	30
Fish oil	2	2	2	2	2	2	2	2	2
Soybean oil	2	2	2	2	2	2	2	2	2
Vit and Min mix ^1,2^	1.9	1.9	1.9	1.9	1.9	1.9	1.9	1.9	1.9
Vit C	0.02	0.02	0.02	0.02	0.02	0.02	0.02	0.02	0.02
Tryptophan	0.5	0.5	0.5	0.5	0.5	0.5	0.5	0.5	0.5
CMC	1	1	1	1	1	1	1	1	1
BHT	0.2	0.2	0.2	0.2	0.2	0.2	0.2	0.2	0.2
Cr2O3	0.5	0.5	0.5	0.5	0.5	0.5	0.5	0.5	0.5
Cellulose	20.1	20.05	20	10.1	10.05	10	0.1	0.05	0
Amylase ^3^	0	0.05	0.1	0	0.05	0.1	0	0.05	0.1
	100	100	100	100	100	100	100	100	100
Proximate composition ^4^ (% dry matter)									
Crude Protein	42.18	41.94	42.69	42.31	42.64	42.21	42.02	41.96	42.64
Crude Lipid	7.16	7.16	7.16	7.33	7.40	7.33	7.13	7.16	7.16
Crude Fiber	6.65	6.43	5.30	6.73	6.30	5.36	4.84	4.75	4.77
Ash	14.50	14.66	15.00	14.66	14.89	15.16	15.33	15.33	15.30
Gross Energy (MJ/Kg)	17.102	17.172	17.242	17.032	16.696	16.562	16.570	16.581	16.608

^1^ The vitamin premix provided the following per kilogram of the diet: Vit. A—10,000,000 IU, Vit. B1—5000 mg, Vit. B2—5000 mg, Vit. B3—6000 mg, Vit. B5—6000 mg, Vit. B6—6000 mg, Vit. C—60,000 mg, Vit. D3—2,000,000 IU, Vit. E—10,000 IU, Vit. H—200 mg. ^2^ The mineral premix provided the following per kilogram of the diet: magnesium—2800 mg, iodine—7.4 mg, iron—7400 mg, copper—1200 mg, manganese—11,600 mg, zinc—9800 mg, chlorides cobalt—4 mg, potassium—100 mg, selenium—4 mg, calcium carbonate—27.25%, phosphorous—7.45 mg, sulfur—0.7 mg, sodium—6 mg, calpan—200 mg, aluminum—1500 mg, choline chloride—10,000 mg. ^3^ Enzyme bioscience Pvt. Ltd., Surat, India. ^4^ Analyzed according to the procedures followed by standard AOAC.

**Table 2:** Growth performance and nutrient utilization of *C. striata* fed different experimental diets.

Treatments	^1^ Final Wt. (g)	^2^ WG (%)	^3^ SGR (%/Day)	^4^ FCR	^5^ PER
C_10_A_0_	27.99 ^ab^ ± 1.56	95.13 ^a^ ± 10.5	0.41 ^ab^ ± 0.03	2.55 ^e^ ± 0.22	0.95 ^a^ ± 0.09
C_10_A_0.05_	31.06 ^bc^ ± 1.68	116.79 ^ab^ ± 12.2	0.48 ^bc^ ± 0.03	1.88 ^cd^ ± 0.13	1.28 ^bc^ ± 0.09
C_10_A_0.1_	34.77 ^de^ ± 0.77	142.72 ^cd^ ± 6.0	0.55 ^de^ ± 0.01	1.78 ^bc^ ± 0.09	1.34 ^bc^ ± 0.07
C_20_A_0_	39.86 ^f^ ± 0.83	178.50 ^e^ ± 5.7	0.64 ^f^ ± 0.01	1.40 ^a^ ± 0.11	1.72 ^e^ ± 0.13
C_20_A_0.05_	35.04 ^de^ ± 1.05	144.24 ^cd^ ± 7.8	0.55 ^de^ ± 0.02	1.61 ^abc^ ± 0.04	1.48 ^cde^ ± 0.04
C_20_A_0.1_	37.30 ^ef^ ± 1.10	160.41 ^de^ ± 7.6	0.59 ^ef^ ± 0.02	1.42 ^ab^ ± 0.03	1.67 ^de^ ± 0.04
C_30_A_0_	27.34 ^a^ ± 0.33	91.28 ^a^ ± 2.2	0.40 ^a^ ± 0.01	2.21 ^de^ ± 0.02	1.08 ^ab^ ± 0.01
C_30_A_0.05_	32.87 ^cd^ ± 1.17	128.43 ^bc^ ± 8.2	0.51 ^cd^ ± 0.02	1.66 ^abc^ ± 0.06	1.44 ^cd^ ± 0.05
C_30_A_0.1_	36.32 ^def^ ± 1.36	154.24 ^cde^ ± 9.5	0.58 ^def^ ± 0.02	1.57 ^abc^ ± 0.18	1.56 ^cde^ ± 0.16
Starch					
10	31.27 ^a^	118.21 ^a^	0.48 ^a^	2.06 ^c^	1.19 ^a^
20	37.40 ^b^	161.05 ^b^	0.59 ^b^	1.47 ^a^	1.62 ^c^
30	32.17 ^a^	124.64 ^a^	0.49 ^a^	1.81 ^b^	1.35 ^b^
Amylase					
0	31.73 ^a^	121.63 ^a^	0.48 ^a^	2.05 ^c^	1.24 ^a^
0.05	32.99 ^a^	129.81 ^a^	0.51 ^a^	1.71 ^b^	1.40 ^b^
0.1	36.13 ^b^	152.46 ^b^	0.57 ^b^	1.59 ^a^	1.52 ^b^
2-way ANOVA *p*-value					
Starch	<0.001	<0.001	<0.001	<0.001	<0.001
Amylase	<0.001	<0.001	<0.001	<0.001	0.004
Starch × Amylase	<0.001	<0.001	<0.001	0.006	0.011

Values are presented as Mean ± SE (n = 3), different superscripts letter in the same column differ significantly (*p* < 0.05). C—Starch; A—amylase. ^1^ Final Wt, final weight (g); ^2^ WG, weight gain %; ^3^ SGR, specific growth rate; ^4^ FCR, feed conversion ratio; ^5^ PER, protein efficiency ratio.

**Table 3:** Whole-body proximate composition of *C. striata* fed different experimental diets (percentage dry matter basis).

Treatments	Moisture	Crude Protein	Crude Lipid	Total Ash
C_10_A_0_	72.13 ± 2.17	17.54 ± 0.06	3.61 ± 0.03	4.15 ± 0.36
C_10_A_0.05_	72.47 ± 2.78	17.48 ± 0.11	3.69 ± 0.03	4.32 ± 0.26
C_10_A_0.1_	72.27 ± 2.77	17.86 ± 0.03	3.58 ± 0.03	4.28 ± 0.34
C_20_A_0_	72.40 ± 2.93	17.67 ± 0.13	3.64 ± 0.04	4.20 ± 0.54
C_20_A_0.05_	72.47 ± 4.24	17.67 ± 0.10	3.60 ± 0.11	4.28 ± 0.33
C_20_A_0.1_	72.53 ± 1.58	17.66 ± 0.13	3.72 ± 0.05	4.20 ± 0.18
C_30_A_0_	72.33 ± 3.20	17.63 ± 0.05	3.47 ± 0.04	4.20 ± 0.28
C_30_A_0.05_	72.47 ± 2.34	17.65 ± 0.10	3.60 ± 0.04	4.22 ± 0.02
C_30_A_0.1_	72.67 ± 5.88	17.65 ± 0.10	3.54 ± 0.06	4.23 ± 0.14
Starch				
10	72.28	17.62	3.62 ^ab^	4.24
20	72.46	17.67	3.65 ^b^	4.22
30	72.48	17.64	3.53 ^a^	4.21
Amylase				
0	72.28	17.61	3.57	4.18
0.05	72.46	17.60	3.62	4.27
0.1	72.48	17.72	3.61	4.23
2 Way ANOVA *p*-value				
Starch	0.997	0.861	0.042	0.991
Amylase	0.997	0.247	0.469	0.934
Starch × Amylase	1.000	0.223	0.246	0.999

Values are presented as Mean ± SE (n = 3), different superscripts letter in the same column differ significantly (*p* < 0.05). C—Starch; A—amylase.

**Table 4:** Apparent digestibility of different experiential diets fed to *C. striata*.

Treatments	Dry Matter (%)	Crude Protein (%)	Crude Lipid (%)	Carbohydrates (%)
C_10_A_0_	57.97 ^bc^ ± 0.04	88.24 ± 0.36	84.59 ± 0.07	81.01 ^c^ ± 0.31
C_10_A_0.05_	59.77 ^d^ ± 0.05	87.76 ± 0.11	85.13 ± 0.09	83.67 ^d^ ± 0.15
C_10_A_0.1_	61.65 ^ef^ ± 0.14	87.73 ± 0.41	84.95 ± 0.09	85.76 ^e^ ± 0.14
C_20_A_0_	57.45 ^b^ ± 0.56	87.67 ± 0.07	85.23 ± 0.06	79.54 ^b^ ± 0.28
C_20_A_0.05_	61.13 ^ef^ ± 0.07	87.82 ± 0.13	85.09 ± 0.09	83.78 ^d^ ± 0.11
C_20_A_0.1_	61.73 ^f^ ± 0.09	88.06 ± 0.09	85.14 ± 0.16	86.47 ^e^ ± 0.26
C_30_A_0_	55.80 ^a^ ± 0.18	87.69 ± 0.14	85.33 ± 0.11	77.16 ^a^ ± 0.49
C_30_A_0.05_	58.29 ^c^ ± 0.27	87.87 ± 0.14	85.15 ± 0.18	79.50 ^b^ ± 0.17
C_30_A_0.1_	61.01 ^e^ ± 0.07	87.96 ± 0.20	85.19 ± 0.25	80.88 ^c^ ± 0.14
Starch				
10	59.79 ^b^	87.91	84.88 ^a^	83.48 ^b^
20	60.10 ^b^	87.84	85.15 ^b^	83.26 ^b^
30	58.36 ^a^	87.84	85.22 ^b^	79.17 ^a^
Amylase				
0	57.07 ^a^	87.86	85.05	79.23 ^a^
0.05	59.73 ^b^	87.81	85.12	82.32 ^b^
0.1	61.46 ^c^	87.91	85.09	84.36 ^c^
2 Way ANOVA *p*-value				
Starch	<0.001	0.909	0.017	<0.001
Amylase	<0.001	0.851	0.806	<0.001
Starch × Amylase	<0.001	0.271	0.085	<0.001

Values are presented as Mean ± SE (n = 3), different superscripts letter in the same column differ significantly (*p* < 0.05). C—Starch; A—amylase.

**Table 5:** Digestive enzyme activity of *C. striata* fed different experimental diets.

Diet	Protease (U/mg Protein)	Lipase (U/mg Protein)	Amylase (U/mg Protein)
C_10_A_0_	1.74 ± 0.01	1.83 ^a^ ± 0.09	0.44 ^ab^ ± 0.11
C_10_A_0.05_	1.71 ± 0.04	1.35 ^abc^ ± 0.08	0.48 ^b^ ± 0.09
C_10_A_0.1_	1.70 ± 0.03	1.03 ^a^ ± 0.04	0.54 ^c^ ± 0.04
C_20_A_0_	1.72 ± 0.03	1.25 ^ab^ ± 0.12	0.43 ^a^ ± 0.02
C_20_A_0.05_	1.74 ± 0.03	1.15 ^ab^ ± 0.18	0.53 ^c^ ± 0.01
C_20_A_0.1_	1.73 ± 0.05	1.28 ^ab^ ± 0.06	0.58 ^d^ ± 0.07
C_30_A_0_	1.71 ± 0.06	0.97 ^a^ ± 0.07	0.42 ^a^ ± 0.12
C_30_A_0.05_	1.69 ± 0.03	1.43 ^abc^ ± 0.06	0.46 ^ab^ ± 0.04
C_30_A_0.1_	1.71 ± 0.05	1.60 ^bc^ ± 0.16	0.52 ^c^ ± 0.01
Starch			
10	1.71	1.40	0.48 ^a^
20	1.72	1.22	0.51 ^b^
30	1.70	1.33	0.46 ^a^
Amylase			
0	1.72	1.35	0.43 ^a^
0.05	1.71	1.31	0.48 ^b^
0.1	1.71	1.30	0.54 ^c^
2 Way ANOVA *p*-value			
Starch	0.715	0.434	<0.000
Amylase	0.939	0.928	<0.000
Starch × Amylase	0.953	0.007	0.092

Values are presented as Mean ± SE (n = 3); different superscript letters in the same column indicate significant differences (*p* < 0.05). C—Starch; A—amylase.

**Table 6:** Metabolic enzyme activity of *C. striata* fed different experimental diets.

Diet	Hexokinase (Milli Units/min/mg Protein)	AST (Serum) (U/L)	ALT (Serum) (U/L)
C_10_A_0_	0.47 ± 0.01	29.96 ± 0.01	1.69 ^c^ ± 0.01
C_10_A_0.05_	0.50 ± 0.03	29.85 ± 0.01	1.65 ^b^ ± 0.02
C_10_A_0.1_	0.53 ± 0.11	29.84 ± 0.02	1.62 ^b^ ± 0.01
C_20_A_0_	0.48 ± 0.04	29.97 ± 0.01	1.72 ^c^ ± 0.01
C_20_A_0.05_	0.50 ± 0.01	29.87 ± 0.04	1.63 ^b^ ± 0.03
C_20_A_0.1_	0.52 ± 0.02	29.85 ± 0.01	1.55 ^a^ ± 0.02
C_30_A_0_	0.48 ± 0.13	30.08 ± 0.03	1.77 ^d^ ± 0.01
C_30_A_0.05_	0.49 ± 0.09	30.01 ± 0.02	1.70 ^c^ ± 0.01
C_30_A_0.1_	0.52 ± 0.02	30.00 ± 0.01	1.62 ^b^ ± 0.02
Starch			
10	0.50	29.88 ^a^	1.65 ^a^
20	0.50	29.89 ^a^	1.63 ^a^
30	0.49	30.02 ^b^	1.69 ^b^
Amylase			
0	0.47 ^a^	30.00 ^b^	1.72 ^c^
0.05	0.50 ^b^	29.90 ^a^	1.65 ^b^
0.1	0.52 ^c^	29.89 ^a^	1.59 ^a^
2 Way ANOVA *p*-value			
Starch	0.869	<0.001	<0.001
Amylase	<0.001	<0.001	<0.001
Starch × Amylase	0.972	0.380	0.008

Values are presented as Mean ± SE (n = 3); different superscript letters in the same column indicate significant differences (*p* < 0.05). C—Starch; A—amylase.

## Data Availability

The data that support the findings of this study are available within the manuscript.

## References

[1] FAO (2024). Global Fisheries and Aquaculture Production Reaches a New Record High.

[2] De Silva S.S., Phuong N.T. (2011). Striped catfish farming in the Mekong Delta, Vietnam: A tumultuous path to a global success. Rev. Aquac..

[3] Aliyu-Paiko M., Hashim R., Shu-Chien A.C. (2010). Influence of dietary lipid/protein ratio on survival, growth, body indices and digestive lipase activity in Snakehead (*Channa striatus*, Bloch 1793) fry reared in re-circulating water system. Aquac. Nutr..

[4] Mustafa A., Widodo M.A., Kristianto Y. (2012). Albumin and zinc content of snakehead fish (*Channa striata*) extract and its role in health. IEESE Int. J. Sci. Technol..

[5] Sinh L.X., Navy H., Pomeroy R.S. (2014). Value chain of snakehead fish in the Lower Mekong Basin of Cambodia and Vietnam. Aquac. Econ. Manag..

[6] Webster C.D., Lim C. (2002). Nutrient Requirements and Feeding of Finfish for Aquaculture.

[7] Sahid N.A., Hayati F., Rao C.V., Ramely R., Sani I., Dzulkarnaen A., Zakaria Z., Hassan S., Zahari A., Ali A.A. (2018). Snakehead consumption enhances wound healing? From tradition to modern clinical practice: A prospective randomized controlled trial. Evid. Based Complement. Altern. Med..

[8] Hemre G.I., Lie Ø., Lied E., Lambertsen G. (1989). Starch as an energy source in feed for cod (*Gadus morhua*): Digestibility and retention. Aquaculture.

[9] Hussain S.M., Khurram F., Naeem A., Shah S.Z.H., Sarker P.K., Naeem E., Arsalan M.Z.U.H., Riaz D., Yousaf Z., Faisal M. (2024). A review on the prospects and potentials of fishmeal replacement with different animal protein sources. Int. Aquat. Res..

[10] Anand G., Bhat I.A., Varghese T., Dar S.A., Sahu N.P., Aklakur M.D., Kumar S., Sahoo S. (2018). Alterations in non-specific immune responses, antioxidant capacities and expression levels of immunity genes in *Labeo rohita* fed with graded level of carbohydrates. Aquaculture.

[11] Honorato C.A., Almeida L.C., Da Silva Nunes C., Carneiro D.J., Moraes G. (2010). Effects of processing on physical characteristics of diets with distinct levels of carbohydrates and lipids: The outcomes on the growth of pacu (*Piaractus mesopotamicus*). Aquac. Nutr..

[12] Rosas C., Cuzon G., Gaxiola G., Arena L., Lemaire P., Soyez C., Van Wormhoudt A. (2000). Influence of dietary carbohydrate on the metabolism of juvenile *Litopenaeus stylirostris*. J. Exp. Mar. Biol. Ecol..

[13] Cheng K., Li P., Zheng S., Zhu X., Ma X., Li X. (2022). Utilization and Metabolism of Dietary Digestible Carbohydrates in Fish. Fish. Sci. Technol. Inf..

[14] Kamalam B.S., Medale F., Panserat S. (2017). Utilisation of dietary carbohydrates in farmed fishes: New insights on influencing factors, biological limitations and future strategies. Aquaculture.

[15] Wang J., Li X., Han T., Yang Y., Jiang Y., Yang M., Xu Y., Harpaz S. (2016). Effects of different dietary carbohydrate levels on growth, feed utilization and body composition of juvenile grouper *Epinephelus akaara*. Aquaculture.

[16] Enes P., Panserat S., Kaushik S., Oliva-Teles A.A. (2006). Effect of normal and waxy maize starch on growth, food utilization and hepatic glucose metabolism in European sea bass (*Dicentrarchus labrax*) juveniles. Comp. Biochem. Physiol. Part A Mol. Integr. Physiol..

[17] Stone D.A. (2003). Dietary carbohydrate utilization by fish. Rev. Fish. Sci..

[18] Paredes J.F., Habte-Tsion M., Riche M., Mejri S., Bradshaw D., Chin L.S., Perricone C., Wills P.S. (2025). Effects of different carbohydrates on growth, hepatic glucose metabolism, and gut microbiome of *Florida pompano* (*Trachinotus carolinus*). Aquaculture.

[19] Zhou C., Ge X., Niu J., Lin H., Huang Z., Tan X. (2015). Effect of dietary carbohydrate levels on growth performance, body composition, intestinal and hepatic enzyme activities, and growth hormone gene expression of juvenile golden pompano, *Trachinotus ovatus*. Aquaculture.

[20] Marandel L., Lepais O., Arbenoits E., Véron V., Dias K., Zion M., Panserat S. (2016). Remodelling of the hepatic epigenetic landscape of glucose-intolerant rainbow trout (*Oncorhynchus mykiss*) by nutritional status and dietary carbohydrates. Sci. Rep..

[21] Hemre G.I., Mommsen T.P., Krogdahl A. (2001). Carbohydrates in fish nutrition: Effects on growth, glucose metabolism and hepatic enzymes. Aquac. Nutr..

[22] Boonanuntanasarn S., Jangprai A., Kumkhong S., Plagnes-Juan E., Veron V., Burel C., Marandel L., Panserat S. (2018). Adaptation of Nile tilapia (*Oreochromis niloticus*) to different levels of dietary carbohydrates: New insights from a long term nutritional study. Aquaculture.

[23] Xiao P., Wu Y., Sha H., Luo X., Zou G., Liang H. (2025). Effects of Different Carbohydrate Levels in Diets on Growth Performance and Muscle Nutritive Value of Ying Carp and Scattered-Scaled Mirror Carp (*Cyprinus carpio*). Aquac. Nutr..

[24] Barton B.A., Iwama G.K. (1991). Physiological changes in fish from stress in aquaculture with emphasis on the response and effects of corticosteroids. Annu. Rev. Fish Dis..

[25] Xu R., Li M., Wang T., Zhao Y.W., Shan C.J., Qiao F., Chen L.Q., Zhang W.B., Du Z.Y., Zhang M.L. (2022). *Bacillus amyloliquefaciens* ameliorates high-carbohydrate diet-induced metabolic phenotypes by restoration of intestinal acetate-producing bacteria in Nile Tilapia. Br. J. Nutr..

[26] Tan Q., Wang F., Xie S., Zhu X., Lei W., Shen J. (2009). Effect of high dietary starch levels on the growth performance, blood chemistry and body composition of gibel carp (*Carassius auratus* var. gibelio). Aquac. Res..

[27] Bedford M. (1996). Enzyme Action-Under the Microscope. Feed Mix.

[28] Batterham E.S. Development of cost-effective diets for the pig industry: How to utilize low quality ingredients to formulate cost-effective diets. Proceedings of the Aquaculture Nutrition Workshop.

[29] Chesson A. (1993). Feed enzyme. Anim. Feed. Sci. Tech..

[30] Ravindran V., Son J.H. (2011). Feed enzyme technology: Present status and future developments. Recent Pat. Food Nutr. Agric..

[31] Kalhoro H., Zhou J., Hua Y., Ng W.K., Ye L., Zhang J., Shao Q. (2018). Soy protein concentrate as a substitute for fish meal in diets for juvenile *Acanthopagrus schlegelii*: Effects on growth, phosphorus discharge and digestive enzyme activity. Aquac. Res..

[32] Maas R.M., Verdegem M.C., Wiegertjes G.F., Schrama J.W. (2020). Carbohydrate utilisation by tilapia: A meta-analytical approach. Rev. Aquac..

[33] Ai Q., Mai K., Zhang W., Xu W., Tan B., Zhang C., Li H. (2007). Effects of exogenous enzymes (phytase, non-starch polysaccharide enzyme) in diets on growth, feed utilization, nitrogen and phosphorus excretion of Japanese seabass, Lateolabrax japonicus. Comp. Biochem. Physiol. Part A Mol. Integr. Physiol..

[34] Farhangi M., Carter C.G. (2007). Effect of enzyme supplementation to dehulled lupin-based diets on growth, feed efficiency, nutrient digestibility and carcass composition of rainbow trout, *Oncorhynchus mykiss* (Walbaum). Aquac. Res..

[35] Lin S., Mai K., Tan B. (2007). Effects of exogenous enzyme supplementation in diets on growth and feed utilization in tilapia, *Oreochromis niloticus* × *O. aureus*. Aquac. Res..

[36] Kumar S., Sahu N.P., Pal A.K., Choudhury D., Mukherjee S.C. (2006). Studies on digestibility and digestive enzyme activities in *Labeo rohita* (Hamilton) juveniles: Effect of microbial α-amylase supplementation in non-gelatinized or gelatinized corn-based diet at two protein levels. Fish Physiol. Biochem..

[37] Stone D.A.J., Allan G.L., Anderson A.A. (2003). Carbohydrate utilization by juvenile silver perch, *Bidyanus bidyanus* (Mitchell). III. The protein-sparing effect of wheat starch-based carbohydrates. Aquac. Res..

[38] Ogunkoya A.E., Page G.I., Adewolu M.A., Bureau D.P. (2006). Dietary incorporation of soybean meal and exogenous enzyme cocktail can affect physical characteristics of faecal material egested by rainbow trout (*Oncorhynchus mykiss*). Aquaculture.

[39] Khalil F., Emeash H. (2018). Behavior and stereotypies of Nile Tilapia (*Oreochromis niloticus*) in response to experimental infection with *Aeromonas hydrophila*. Aquat. Sci. Eng..

[40] Carter C.G., Houlihan D.F., Buchanan B., Mitchell A.I. (1994). Growth and feed utilization efficiencies of seawater Atlantic salmon, *Salmo salar* L., fed a diet containing supplementary enzymes. Aquac. Res..

[41] Association of Official Analytical Chemists (AOAC) (2010). Official Methods of Analysis of AOAC International.

[42] Yigit M., Sahinyilmaz M., Acar Ü., Kesbic O., Yilmaz S., Bulut M., Gürses K., Maita M. (2018). Evaluation of dietary protein level in practical feed for twoband bream Diplodus vulgaris. N. Am. J. Aquac..

[43] Ranjan A., Sahu N.P., Deo A.D., Kumar H.S., Kumar S., Jain K.K. (2018). Comparative evaluation of fermented and non-fermented de-oiled rice bran with or without exogenous enzymes supplementation in the diet of *Labeo rohita* (Hamilton, 1822). Fish Physiol. Biochem..

[44] Drapeau G., Lorand B.L. (1974). Protease from *Staphylococcus aureus*. Methods in Enzymology.

[45] Cherry I.S., Crandall L.A. (1932). The specificity of pancreatic lipase: Its appearance in the blood after pancreatic injury. Am. J. Physiol. Leg. Content.

[46] Rick W., Stegbauer H.P. (1974). α-Amylase measurement of reducing groups. Methods of Enzymatic Analysis.

[47] Easterby J.S., O’Brien M.J. (1973). Purification and properties of pig-heart hexokinase. Eur. J. Biochem..

[48] Shiau S.Y., Liang H.S. (1994). Carbohydrate utilization and digestibility by tilapia *Oreochromis niloticus* × *O. aureus* are affected by chromic oxide inclusion in the diet. J. Nutr..

[49] Kumar S., Sahu N.P., Pal A.K., Choudhury D., Yengkokpam S., Mukherjee S.C. (2005). Effect of dietary carbohydrate on haematology, respiratory burst activity and histological changes in *L. rohita* juveniles. Fish Shellfish Immunol..

[50] Goda A.M.A., Mabrouk H.A.H.H., Wafa M.A.E.H., El-Afifi T.M. (2012). Effect of using baker’s yeast and exogenous digestive enzymes as growth promoters on growth, feed utilization and hematological indices of Nile tilapia, *Oreochromis niloticus* fingerlings. J. Agric. Sci. Technol..

[51] Li J., Li J., Wu T. (2009). Effects of non-starch polysaccharides enzyme, phytase and citric acid on activities of endogenous digestive enzymes of tilapia (*Oreochromis niloticus* × *Oreochromis aureus*). Aquac. Nutr..

[52] Zhang L., Mai K.S., Ai Q.H., Tan B.P. (2006). Effects of phytase and non-starch polysaccharide enzyme supplementation in diets on growth and digestive enzyme activity in large yellow croaker, *Pseudosciaena crocea* R. Period. Ocean Univ. China.

[53] Hlophe-Ginindza S.N., Moyo N.A., Ngambi J.W., Ncube I. (2016). The effect of exogenous enzyme supplementation on growth performance and digestive enzyme activities in *Oreochromis mossambicus* fed kikuyu-based diets. Aquac. Res..

[54] Zhu D., Wen X., Li S., Xuan X., Li Y. (2016). Effects of exogenous non-starch polysaccharide-degrading enzymes in diets containing *Gracilaria lemaneiformis* on white-spotted snapper *Lutjanus stellatus* Akazaki. Aquac. Int..

[55] Song H.L., Tan B.P., Chi S.Y., Liu Y., Chowdhury M.K., Dong X.H. (2017). The effects of a dietary protease-complex on performance, digestive and immune enzyme activity, and disease resistance of *Litopenaeus vannamei* fed high plant protein diets. Aquac. Res..

[56] Drew M.D., Racz V.J., Gauthier R., Thiessen D.L. (2005). Effect of adding protease to coextruded flax: Pea or canola: Pea products on nutrient digestibility and growth performance of rainbow trout (*Oncorhynchus mykiss*). Anim. Feed Sci. Technol..

[57] Jobling M. (1994). Fish Bioenergetics.

[58] Liu Q.Z., Zhang H., Dai H.Q., Zhao P., Mao Y.F., Chen K.X., Chen Z.X. (2021). Inhibition of starch digestion: The role of hydrophobic domain of both α-amylase and substrates. Food Chem..

[59] Kumar S., Sahu N.P., Pal A.K., Choudhury D., Mukherjee S.C. (2006). Non-gelatinized corn supplemented with α-amylase at sub-optimum protein level enhances the growth of *Labeo rohita* (Hamilton) fingerlings. Aquac. Res..

[60] Dalsgaard J., Verlhac V., Hjermitslev N.H., Ekmann K.S., Fischer M., Klausen M., Pedersen P.B. (2012). Effects of exogenous enzymes on apparent nutrient digestibility in rainbow trout (*Oncorhynchus mykiss*) fed diets with high inclusion of plant-based protein. Anim. Feed Sci. Technol..

[61] Hassaan M.S., Mohammady E.Y., Soaudy M.R., Abdel Rahman A.A. (2019). Exogenous xylanase improves growth, protein digestibility and digestive enzymes activities in Nile tilapia, *Oreochromis niloticus*, fed different ratios of fish meal to sunflower meal. Aquac. Nutr..

[62] Onderci M., Sahin N., Sahin K., Cikim G., Aydin A., Ozercan I., Aydin S. (2006). Efficacy of supplementation of α-amylase-producing bacterial culture on the performance, nutrient use, and gut morphology of broiler chickens fed a corn-based diet. Poult. Sci..

[63] Breck J.E. (2014). Body composition in fishes: Body size matters. Aquaculture.

[64] Haider M.S., Ashraf M., Azmat H., Khalique A., Javid A., Atique U., Zia M., Iqbal K.J., Akram S. (2016). Nutritive evaluation of fish acid silage in *Labeo rohita* fingerlings feed. J. Appl. Anim. Res..

[65] Sajjadi M., Carter C.G. (2004). Effect of phytic acid and phytase on feed intake, growth, digestibility and trypsin activity in Atlantic salmon (*Salmo salar* L.). Aquac. Nutr..

[66] Ng W.K., Chong K.K. (2002). The nutritive value of palm kernel and the effect of enzyme supplementation in practical diets for red hybrid tilapia (*Oreochromis* sp.). Asian Fish. Sci..

[67] Liu H., Pan L., Shen J., Tan B., Dong X., Yang Q., Chi S., Zhang S. (2023). Effects of carbohydrase supplementation on growth performance, intestinal digestive enzymes and Flora, glucose metabolism enzymes, and glut2 gene expression of hybrid grouper (*Epinephelus fuscoguttatus*♀ × *E. lanceolatus*♂) fed different CHO/L ratio diets. Metabolites.

[68] Govoni J.J., Boehlert G.W., Watanabe Y. (1986). The physiology of digestion in fish larvae. Environ. Biol. Fishes.

[69] Hidalgo M.C., Urea E., Sanz A. (1999). Comparative study of digestive enzymes in fish with different nutritional habits. Proteolytic and amylase activities. Aquaculture.

[70] Li X.Q., Chai X.Q., Liu D.Y., Kabir Chowdhury M.A., Leng X.J. (2016). Effects of temperature and feed processing on protease activity and dietary protease on growths of white shrimp, *Litopenaeus vannamei*, and tilapia, *Oreochromis niloticus* × *O. aureus*. Aquac. Nutr..

[71] Mohapatra M., Sahu N.P., Chaudhari A. (2003). Utilization of gelatinized carbohydrate in diets of *Labeo rohita* fry. Aquac. Nutr..

[72] Ali M.Z., Jauncey K. (2004). Optimal dietary carbohydrate to lipid ratio in African catfish *Clarias gariepinus* (Burchell 1822). Aquac. Int..

[73] Jiang T.T., Feng L., Liu Y., Jiang W.D., Jiang J., Li S.H., Tang L., Kuang S.Y., Zhou X.Q. (2014). Effects of exogenous xylanase supplementation in plant protein-enriched diets on growth performance, intestinal enzyme activities and microflora of juvenile Jian carp (*Cyprinus carpio* var. J ian). Aquac. Nutr..

[74] Magalhães R., Lopes T., Martins N., Díaz-Rosales P., Couto A., Pousão-Ferreira P., Oliva-Teles A., Peres H. (2016). Carbohydrases supplementation increased nutrient utilization in white seabream (*Diplodus sargus*) juveniles fed high soybean meal diets. Aquaculture.

[75] Zhou Y., Yuan X., Liang X.F., Fang L., Li J., Guo X., Bai X., He S. (2013). Enhancement of growth and intestinal flora in grass carp: The effect of exogenous cellulase. Aquaculture.

[76] Tian J., Lu X., Jiang M., Wu F., Liu W., Yu L., Wen H. (2020). AMPK activation by dietary AICAR affects the growth performance and glucose and lipid metabolism in juvenile grass carp. Aquac. Nutr..

[77] Gilannejad N., Martínez-Rodríguez G., Moyano F.J., Yúfera M. (2018). Effects of Different Feeding Protocols on Daily Proteolitic Enzyme Activity in Gilthead Seabream Juveniles.

[78] Walton M.J., Cowey C.B. (1982). Aspects of intermediary metabolism in salmonid fish. Comp. Biochem. Physiol. Part B Comp. Biochem..

[79] Panserat S., Capilla E., Gutierrez J., Frappart P.O., Vachot C., Plagnes-Juan E., Aguirre P., Breque J., Kaushik A.S. (2001). Glucokinase is highly induced and glucose-6-phosphatase poorly repressed in liver of rainbow trout (*Oncorhynchus mykiss*) by a single meal with glucose. Comp. Biochem. Physiol. Part B Biochem. Mol. Biol..

[80] Furuichi M., Yone Y. (1982). Availability of carbohydrate in nutrition of carp and red sea bream. Bull. Jpn. Sot. Sci. Fish..

[81] Furuichi M., Yone Y. (1982). Effect of insulin on blood sugar levels of fishes. Bull. Jpn. Sot. Sci. Fish..

[82] Wilson R.P., Poe W.E. (1987). Apparent inability of channel catfish to utilize dietary mono-and disaccharides as energy sources. J. Nutr..

[83] Dandin E., Üstündağ Ü.V., Ünal İ., Ateş-Kalkan P.S., Cansız D., Beler M., Ak E., Alturfan A.A., Emekli-Alturfan E. (2022). Stevioside ameliorates hyperglycemia and glucose intolerance, in a diet-induced obese zebrafish model, through epigenetic, oxidative stress and inflammatory regulation. Obes. Res. Clin. Pract..

[84] Capilla E., Médale F., Navarro I., Panserat S., Vachot C., Kaushik S., Gutiérrez J. (2003). Muscle insulin binding and plasma levels in relation to liver glucokinase activity, glucose metabolism and dietary carbohydrates in rainbow trout. Regul. Pept..

[85] Borrebaek B., Waagbø R., Christophersen B., Tranulis M.A., Hemre G.I. (1993). Adaptable hexokinase with low affinity for glucose in the liver of Atlantic salmon (*Salmo salar*). Comp. Biochem. Physiol. Part B Comp. Biochem..

[86] Gao X.Q., Fei F., Huo H.H., Huang B., Meng X.S., Zhang T., Liu B.L. (2020). Impact of nitrite exposure on plasma biochemical parameters and immune-related responses in *Takifugu rubripes*. Aquat. Toxicol..

[87] Sheikh Z.A., Ahmed I. (2019). Impact of environmental changes on plasma biochemistry and hematological parameters of Himalayan snow trout, *Schizothorax plagiostomus*. Comp. Clin. Pathol..

[88] Hilton J.W., Atkinson J.L. (1982). Response of rainbow trout (*Salmo gairdneri*) to increased levels of available carbohydrate in practical trout diets. Br. J. Nutr..

[89] Yang J.L., Chen H.C. (2003). Serum metabolic enzyme activities and hepatocyte ultrastructure of common carp after gallium exposure. Zool. Stud..

[90] Zhai S.W., Shi Q.C., Chen X.H. (2016). Effects of dietary surfactin supplementation on growth performance, intestinal digestive enzymes activities and some serum biochemical parameters of tilapia (*Oreochromis niloticus*) fingerlings. Ital. J. Anim. Sci..

[91] Amin K.A., Hashem K.S. (2012). Deltamethrin-induced oxidative stress and biochemical changes in tissues and blood of catfish (*Clarias gariepinus*): Antioxidant defense and role of alpha-tocopherol. BMC Vet. Res..

